# Use of Corrected QT Cutoffs Derived from Biological Variation to Predict Adverse Events Due to Antipsychotic Drugs: Case Report

**DOI:** 10.5811/cpcem.48764

**Published:** 2026-08-05

**Authors:** Alan H.B. Wu, Melissa Alamillo, Kayla Kendrick

**Affiliations:** * University of California, San Francisco, Department of Laboratory Medicine, San Francisco, California; † University of California, San Francisco, Department of Emergency Medicine, San Francisco, California

**Keywords:** electrocardiogram, QTc interval, biological variation, case report

## Abstract

**Introduction:**

The use of antipsychotic drugs can prolong the corrected QT (QTc) interval of the electrocardiogram and cause a life-threatening ventricular arrhythmia. There is no consensus as to what is considered normal or what cutoff indicates QTc prolongation. However, recent literature has described the biological variation of the QTc interval from healthy subjects, with researchers concluding that the best approach at establishing a normal range is to determine an individual baseline interval during health.

**Case Report:**

A baseline QTc interval (460 milliseconds) had been determined for a 42-year-old female with a history of schizophrenia and depression who was prescribed antipsychotic drugs including escitalopram, olanzapine, haloperidol, clonazepam and divalproex over the course of four years. Over that time frame, she was admitted on 20 occasions with chest pain, but her QTc interval was at or above her baseline level. Acute coronary syndrome was ruled out for each episode. Her medication history was not altered or discontinued until her QTc was greatly prolonged at 530 milliseconds several years later. Fortunately, she did not suffer torsades de pointes.

**Conclusion:**

This case illustrates how results from biological variation studies and a personalized reference level can be helpful to alert physicians earlier of the presence of a potentially toxic condition.

## INTRODUCTION

Patients receiving care from psychiatry services are frequently prescribed antidepressant medications to manage their conditions. These agents are associated with side effects that range from mild symptoms such as dry mouth, sedation, weight gain, and extrapyramidal effects^1^,^2^ to more severe complications such as malignant neuroleptic syndrome.^3^ Antidepressant use has also been linked to cardiovascular effects, with manifestations such as electrocardiogram (ECG) abnormalities, including corrected QT (QTc) interval prolongation and the development of torsades de pointes, a form of ventricular arrhythmia.^4^

In a previous study, we evaluated the biological variation of quantitative parameters derived from the ECG, specifically the QTc interval (corrected for heart rate using Bazett formula) and the QRS complex duration.^5^ We measured ECGs in duplicate on healthy subjects once per week for four weeks. This design allowed us to demonstrate low intra-individual variability and high inter-individual variability for both parameters. Population-based reference intervals for the QTc are wide. Our observations demonstrated that an individual who typically exhibits QTc values at the lower end of the population range could experience a substantial increase—one that may be clinically significant—yet still fall within the conventional “normal” limits. A more meaningful approach is to establish an individual’s baseline during a period of health and interpret future results in relation to this personal reference range. Biological variation studies also yield reference change values, which define the degree of change from baseline required for a new result to be considered statistically significant.^6^ For the QTc interval, the reference change values were calculated at 7.6% for males and 6.4% for females.^5^

In the absence of individualized reference intervals, many studies have proposed population-based QTc cutoffs to identify clinically significant prolongation associated with antipsychotic drug use. However, these thresholds vary widely. Huffman et al classify QTc values > 450 milliseconds (msec) as “borderline” and those exceeding 500 msec as high risk for torsades de pointes.^7^ Dietle et al recommend gender-specific thresholds of 440 msec for men and 460 msec for women.^8^ The American Heart Association defines QTc prolongation as > 470 msec in men and > 480 msec in women,^4^ while the British Heart Rhythm Society also adopts a 470-msec cutoff for women.^9^ This lack of consensus complicates clinical decision-making and reduces the utility of QTc monitoring as a reliable early warning sign of drug-induced cardiac toxicity.

## CASE REPORT

This case began in the summer of 2019, when a 42-year-old Black female presented to the emergency department (ED) with her first episode of sharp, episodic mid-sternal chest pain accompanied by nausea but without shortness of breath or diaphoresis. The chart abstractors were not blinded to the study hypothesis. Her medical history included hypertension, deep vein thrombosis/pulmonary embolus, post-traumatic stress disorder, and psychosis. She was not obese, smoked half a pack of cigarettes daily, and had a normal lipid profile.

Over the next five years, she presented to the ED 123 times and was admitted to the hospital 18 times. Her complaints included falls, drug overdoses, headaches, abdominal pain, and suicidal ideation. She presented with chest pain on 20 separate occasions. Due to her psychiatric conditions, she was treated with antipsychotic medications including escitalopram, haloperidol, olanzepine, clonazepam, and divalproex. During some of these episodes, there were notes of suicidal ideation and intentional opioid overdoses. However, there was no mention of overdose by antipsychotic medications.

Standard 12-lead ECG recordings were obtained on 46 occasions, both to monitor her response to these medications and to evaluate her chest pain. None of these ECGs demonstrated evidence of acute myocardial ischemia during the episodes of chest pain admissions. Serial blood samples were collected and tested for high-sensitivity cardiac troponin I, all of which were negative, with most results near the assay’s limit of quantification (Centaur and Attelica IM, Siemens Healthineers). Acute myocardial infarction was ruled out in all 20 presentations of chest pain. We tracked her QTc interval (Bazett formula), which ranged from 409–530 msec. At no time did her ECGs reveal torsades de pointes. This case report was not reviewed by the institutional review board. However, the patient provided written consent allowing the use of her medical information and medications history.

*CPC-EM Capsule*

What do we already know about this clinical entity?

*Antidepressant drugs can prolong the correct QT (QTc) interval on the electrocardiogram. A previous study showed that an increase of 8% from baseline is medically significant.*

What makes this presentation of disease reportable?

*This case shows that a patient given antidepressant medications may be toxic even if the QTc interval is below published cutoffs from national guidelines.*

What is the major learning point?

*The use of a personalized QTc cutoff provides a better assessment of risk for cardiac complications than using a generalized cutoff established by guidelines.*

How might this improve emergency medicine practice?

*For drugs that prolong the QTc interval, detection of risks is best achieved by comparing current results against prior readings during outpatient visits.*

Figure A displays four QTc measurements recorded from June–October 2020, just after her first ED episode of chest pain. During this period, she had no further ED admissions, had routine ECGs with stable QTc results, and was prescribed anti-psychotic medications. Electrocardiogram results prior to her initial diagnosis and initiation of these medications were not available. However, since the QTc interval was stable during this three-month period of time, we used these data to establish the patient’s baseline. The mean QTc was 432 (4.9) msec, coefficient of variance of 1.1%. Based on the reference change value for the QTc interval in females (6.4%), a personalized QTc cutoff for this patient would be 460 msec—positioned at the lower end of thresholds recommended by some international groups.

Figures B-D1 show the patient’s QTc measurements during various episodes of chest pain. In November 2019, she presented to the ED with her first episode of chest pain with QTc prolongation of 466 msec. One year later, she had another episode of chest pain with a QTc of 442 msec, which was below her individualized cutoff, but had increased from 419 msec recorded one week earlier. In February 2021, she experienced a third episode of chest pain, with a QTc measurement near her personalized cutoff five days prior (Figure B).

Her QTc intervals remained normal for the next year. In April 2024, the patient experienced two episodes of chest pain. During the first episode, her QTc was 472 msec, preceded a few days earlier by an even higher QTc of 487 msec. (The ECG from the 4/29/24 episode was missing from the medical record). Her QTc intervals remained elevated for the next several months (470 and 495 msec) (Figure C).

Beginning in November 2024, she had four additional ED presentations for chest pain. Many of her ECGs during this period demonstrated prolonged QTc intervals, including one exceptional reading of 530 msec. Fortunately, none of the tracings showed a pattern consistent torsades de pointes (Figure D).

The table below shows the antipsychotic medications given to her after various times before an ED presentation for chest pain, and the discharge medications from several of her ED chest pain episodes. While her medication regimen varied slightly from 2019–2024, the only change in response to the QTc results was the discontinuance of escitalopram on July 1, 2024 (QTc = 470 msec) and removal of divalprolex and olanzapine on November 27, 2024 (QTc = 530 msec). None of the other increased QTc values reached the attention of the attending medical staff to the extent that they made a change in medication.

## DISCUSSION

Corrected QT prolongation is a well-documented adverse effect of many antipsychotic and psychotropic medications,^10^–^13^ including many of the medications prescribed to this patient. Haloperidol and quetiapine have consistently been associated with QTc prolongation, as identified in the review by Nielsen et al.^10^ Olanzapine, while considered lower risk, has also been associated with modest QTc increases.^11^ In addition, case reports suggest that both hydroxyzine^12^ and buspirone may contribute to QTc prolongation,^13^ though data for the latter are limited. Other non-psychotic drugs that can cause long QT syndrome include nonsteroidal antiinflammatory drugs (eg, ketolac), opioids, anticonvulsive drugs (eg, lamotrigine), muscle relaxants (eg, tizanidine), antiemetics (including metoclopramide), and proton-pump inhibitors and diuretics that potentiate electrolyte loss.^14^ Prolongation of the QT interval increases the risk of ventricular arrhythmias, including the potentially fatal torsades de pointes.^15^ Beyond psychotropics, this patient was also prescribed methadone for chronic pain management—an opioid with well-established QT-prolonging properties and a recognized risk of precipitating torsades de pointes, particularly at higher doses or when combined with other QTc-prolonging agents.^16^ While this patient’s ECGs did not reveal torsades de pointes or other malignant arrhythmias, her QTc intervals repeatedly exceeded both population-based and individualized thresholds. These findings raise important considerations for ongoing monitoring and personalized risk assessment, especially in patients on complex psychotropic regimens.

What makes this case particularly instructive is the dynamic pattern of QTc changes observed in the setting of frequent medication adjustments and recurrent ED visits. While most clinical protocols rely on population-based cutoffs, this case demonstrates the potential benefit of personalized QTc monitoring. Using the concept of reference change values, we established a patient-specific QTc threshold of 460 msec. Several of the patient’s QTc values exceeded this threshold, reflecting a meaningful physiologic deviation rather than normal variability. This approach may help clinicians distinguish between benign and concerning QTc prolongation, especially in medically and psychiatrically complex patients.

Importantly, QTc prolongation itself does not cause chest pain. However, delayed ventricular repolarization can predispose to subclinical arrhythmias or autonomic instability, which may manifest as palpitations, lightheadedness, or presyncope—symptoms that patients may subjectively describe as chest discomfort.^17^ In patients with psychiatric comorbidities, heightened interoceptive sensitivity and anxiety can further amplify or misinterpret these sensations as chest pain. While no malignant arrhythmias were captured during this patient’s ED visits, the temporal relationship between her QTc prolongation and chest pain presentations raises the possibility that transient electrophysiologic changes may have contributed to her symptoms.

This case highlights the clinical utility of establishing a personalized QTc threshold for patients on long-term antipsychotic therapy. In this patient, ECGs exceeding her individualized reference interval frequently preceded episodes of chest pain. Although QTc prolongation was not the direct cause of her symptoms, it may have served as a physiologic marker of drug-related cardiac stress or arrhythmic potential. Recognition of these early warning signs could prompt timely medication reassessment or dosage modification, potentially preventing future ED visits and reducing the risk of serious cardiac events. If a patient presents to the ED with symptoms suggestive of antipsychotic drug effects, the use of a personalized QTc cutoff could be used to determine whether the finding is indicative of drug-induced prolongation. This is an important finding given that these drugs can cause sudden cardiac death.^18^ Furthermore, QTc prolongation is associated with a previous myocardial infarction^19^ and with short- and long-term major adverse cardiac events.^20^ If the result is within personalized limits, it would be suggestive of some other etiology. The presentation of chest pain as seen in this case is more typically the result of acute coronary syndromes.

## LIMITATIONS

This case report has several important limitations. First is the absence of the patient’s true baseline QTc interval prior to the initiation of her antipsychotic medications. While this is usually conducted during a period of health, serial ECG tracings are not routinely conducted in the general population. Given this limitation, the best manner for implementing a personalized cutoff is for attending physicians and psychiatrists to establish a baseline interval during a period of QTc stability over months or years and to compute a mean and adding an appropriate gender specific reference change interval based on published reports.^5^ This personalized QTc cutoff indicating prolongation should be made available to the patient’s medical record so that when a new ECG is conducted, eg, within the ED or elsewhere, proper interpretation can be made by the care team.

A second limitation is that not all ED visits for chest pain included comprehensive documentation of symptom quality or associated features such as palpitations, lightheadedness, or syncope. This limited our ability to correlate QTc prolongation with potential arrhythmic symptoms. Third, because the data were collected retrospectively through chart review, it is impossible to confirm whether the patient was taking her medications as prescribed. Medication adherence in patients with psychiatric comorbidities is often variable, and drug levels were not consistently available. Fourth, the patient received care across multiple hospital systems, which made it difficult to determine the precise timing of medication changes, dosages, or the initiation and discontinuation of specific antipsychotics. These factors limit the strength of causal inference between drug regimen and QTc fluctuations.

## CONCLUSION

From a systems perspective, this case underscores the importance of integrated care for patients with intersecting psychiatric and cardiovascular risks. The patient’s care was fragmented across multiple institutions, complicating longitudinal ECG and medication tracking. Coordinated monitoring using shared electronic health records, individualized QTc thresholds, and careful medication reconciliation may help mitigate the cumulative risk of drug-induced cardiac toxicity.

## Figures and Tables

**Figure A:**
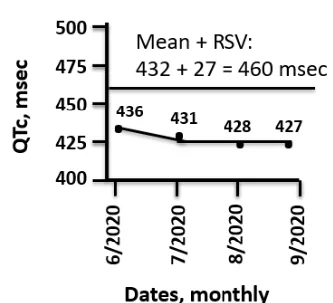
Corrected QT results during a well-controlled disease period. The mean of four readings was 432 milliseconds (msec). Adding the reference change value (6.4% or 27 msec), the individualized cutoff point for QTc prolongation is 460 msec. *QTc*, corrected QT; *msec*, milliseconds; *RCV*, reference change value.

**Figure B:**
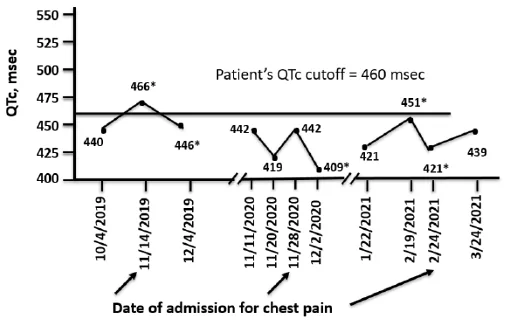
Corrected QT measurements during periods of chest pain from October 2019–March 2021. The line denotes this patient’s cutoff of 460 milliseconds and the * denotes those values that exceed that reference change value. Note: The time axis is not to scale, and the QTc results between readings are not necessarily linear. *msec*, milliseconds; *QTc*, corrected QT.

**Figure C:**
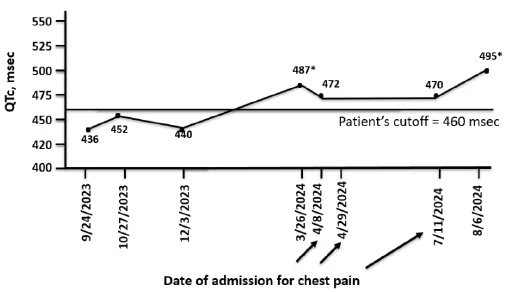
Corrected QT measurements during periods of chest pain from September 2023–August 2024. The line denotes this patient’s cutoff of 460 msec and those values above it that exceed that reference change value. Note: The time axis is not to scale, and the QTc results between readings are not necessarily linear. *msec*, milliseconds; *QTc*, corrected QT.

**Figure D:**
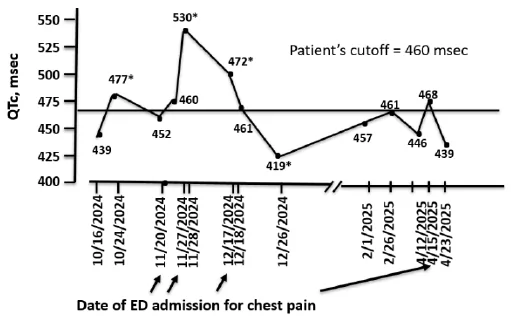
Corrected QT measurements during periods of chest pain from October 2024–April 2025. The line denotes this patient’s cutoff of 460 milliseconds, and those values above that line exceed that reference change value. Note: The time axis is not to scale and the QTc results between readings are not necessarily linear. *QTc*, corrected QT; *msec*, milliseconds.

**Table:** Summary of antipsychotic medications prescribed before and after emergency department episodes of chest pain.

Prior visit	Medications	ED with chest pain	Medication change	Status
11/13/2019	Escitalopram, 10 mg Haloperiodol, 5 mg Olanzapine, 20 mg	11/14/2019	Escitalopram, 10 mg Haloperidol, 5 mg Olalnzapine 20 mg	No change
11/20/2020	Escitalopram, 10 mg Olanzapine, 20 mg	11/28/2020	Escitalopram, 10 mg Olanzapine, 20 mg	No change
2/19/2021	Escitalopram, 10 mg Olanzepine, 20 mg Clonazepam, 1.5 mg	2/24/2021	Escitalopram, 10 mg Olanzepine, 20 mg Clonazepam, 1.5 mg	No change
3/15/2024	Escitalopram, 5 mg	3/26/2024	Escitalopram, 5 mg Divalproex, 1750 mg	Divalproex added
4/8/2024	Escitalopram 5 mg Divalproex, 1750 mg	7/1/2024	Divalproex, 1750 mg	Escitalopram removed
10/24/2024	Olanzepine, 10 mg	11/27/2024	No antipsychotic drugs	Divalproex, Olanzapine removed
11/18/2024	No antipsychotic drugs	12/17/2024	No antipsychotic drugs	No change

*ED*, Emergency Department; *mg*, milligram.
